# Anxiolytic-like Effect of Quercetin Possibly through GABA Receptor Interaction Pathway: In Vivo and In Silico Studies

**DOI:** 10.3390/molecules27217149

**Published:** 2022-10-22

**Authors:** Md. Shahazul Islam, Rajib Hossain, Taukir Ahmed, Md. Mizanur Rahaman, Khattab Al-Khafaji, Rasel Ahmed Khan, Chandan Sarkar, Mehedi Hasan Bappi, Edlane Martins de Andrade, Isaac Moura Araújo, Henrique Douglas Melo Coutinho, Grażyna Kowalska, Radosław Kowalski, Muhammad Asif Hanif, Muhammad Torequl Islam

**Affiliations:** 1 Department of Pharmacy, Life Science Faculty, Bangabandhu Sheikh Mujibur Rahman Science and Technology University, Gopalganj 8100, Bangladesh; 2 College of Dentistry, The University of Mashreq, Baghdad 10022, Iraq; 3 Pharmacy Discipline, Life Science School, Khulna University, Khulna 9280, Bangladesh; 4 CECAPE—College of Dentistry, Av. Padre Cícero 3917, São José 63024-015, Brazil; 5 Department of Biological Chemistry, Regional University of Cariri—URCA, Crato 63105-000, Brazil; 6 Department of Tourism and Recreation, University of Life Sciences in Lublin, 15 Akademicka Str., 20-950 Lublin, Poland; 7 Department of Analysis and Food Quality Assessment, University of Life Sciences in Lublin, 8 Skromna Str., 20-704 Lublin, Poland; 8 Nano and Biomaterials Lab, Department of Chemistry, University of Agriculture, Faisalabad 38040, Pakistan

**Keywords:** anxiety, quercetin, GABAergic system, in vivo study, molecular docking

## Abstract

Scientific evidence suggests that quercetin (QUR) has anxiolytic-like effects in experimental animals. However, the mechanism of action responsible for its anxiolytic-like effects is yet to be discovered. The goal of this research is to assess QUR’s anxiolytic effects in mouse models to explicate the possible mechanism of action. After acute intraperitoneal (i.p.) treatment with QUR at a dose of 50 mg/kg (i.p.), behavioral models of open-field, hole board, swing box, and light–dark tests were performed. QUR was combined with a GABAergic agonist (diazepam) and/or antagonist (flumazenil) group. Furthermore, in silico analysis was also conducted to observe the interaction of QUR and GABA (α5), GABA (β1), and GABA (β2) receptors. In the experimental animal model, QUR had an anxiolytic-like effect. QUR, when combined with diazepam (2 mg/kg, i.p.), drastically potentiated an anxiolytic effect of diazepam. QUR is a more highly competitive ligand for the benzodiazepine recognition site that can displace flumazenil (2.5 mg/kg, i.p.). In all the test models, QUR acted similar to diazepam, with enhanced effects of the standard anxiolytic drug, which were reversed by pre-treatment with flumazenil. QUR showed the best interaction with the GABA (α5) receptor compared to the GABA (β1) and GABA (β2) receptors. In conclusion, QUR may exert an anxiolytic-like effect on mice, probably through the GABA-receptor-interacting pathway.

## 1. Introduction

The World Health Organization (WHO) defines mental health as a state of emotional and psychological well-being in which the person can use their cognitive and emotional abilities, function in society, respond to the demands of daily life, and establish satisfactory and mature relationships with others to participate constructively in social change and adapt to external conditions and internal conflicts [1]. According to Janet anxiety is an objectless fear operating by delay as an embarrassing experience “of imminent and indefinite danger, like a tense state of expectation” [2]. Anxiety is characterized by a diffuse, unpleasant, vague, fearful, or anxious feeling, accompanied by vegetative symptoms: headache, sweating, palpitations, tachycardia, and gastric discomfort [3,4]. Therefore, it consists of two components, one physiological and one psychological, the individual being aware of the existence of both [5]. Anxiety affects thinking, perception, and learning and can distort perceptions, decreasing the power of concentration, associative memory, and evocation. Another important aspect is its effect on attention selectivity [6]. Thus, an anxious person will select certain surrounding things or events and will exaggerate the importance of others in trying to justify their anxiety in response to a frightening situation. Due to the reduction in GABA conduction, anxiety is shown in humans [5]. Cortical GABA_A_ receptor deficiency corresponds with the intensity of anxiety symptoms in panic disorder. A partial GABA_A_ receptor deficiency in γ2 subunit heterozygous mice causes anxiety (which can be corrected with a sustained antidepressant or diazepam) [1,2]. GABA_A_ receptor variant α2 mediates anxiolytic activities, although subtypes α3 and α5 mediate myorelaxant effects [5,6].

Benzodiazepines (BDZs) are used for treating anxiety, but they come with a long list of adverse effects, including drowsiness, muscular relaxation, forgetfulness, and the possibility of addiction. Although anxiolytic drugs are one of the important pharmacological classes in treatment of anxiety, clinical studies have shown that selective serotonin reuptake inhibitors (SSRI) are also effective in treating anxiety [7]. Serotonin is a neurotransmitter (a messenger chemical that carries signals between nerve cells in the brain). It is considered to have an influence on mood, emotion, and sleep. After carrying a message, serotonin is usually reabsorbed by nerve cells (known as reuptake). SSRIs work by blocking (inhibiting) reuptake, which means that more serotonin is available to transmit additional messages between nearby nerve cells.

These SSRIs may exacerbate the symptoms of anxiety after the first administration and, although many preclinical pharmacological studies have been performed in the last 30 years, the exact mechanisms by which SSRIs exert short- and long-term anti-anxiety effects are not yet known [8]. Thus, none of these drugs can be said to be safe, stimulating researchers to conduct more studies with new potentially anxiolytic drugs that are more effective, better tolerated, and have fewer side effects [3]. Due to the undesirable side effects of benzodiazepines and SSRIs, attempts have been made to develop new adjuvant and complementary therapies based on natural bioactive compounds, potentially effective in management of anxiety [9]. Quercetin (QUR) (IUPAC name: 2-(3,4-dihydroxyphenyl)-3,5,7-trihydroxychromen-4-one) is a bitter-tasting plant flavanol from the flavonoid family of polyphenols that may be found in fruits, vegetables, leaves, and grains, such as red onions and kale, and is mostly utilized in nutritional supplements, drinks, and meals [10]. It has been reported that QUR has many important biological properties, including antioxidant [11], anti-inflammatory [12], neuroprotective [13], and anticancer [14]. Cumulative reports suggest that QUR has anxiolytic-like effects in experimental animals [15,16,17]. Studies suggest that QUR could interact with the GABA-α5 receptor for reducing seizures [18] and with GABA receptor β1 and β3 subunits for its anti-epileptic effect [19] in experimental animals.

To date, none of the studies published in the literature have suggested the possible mechanisms behind the potential anxiolytic effect of QUR. In light of these aspects, the current study aimed to assess the potential anxiolytic effect of QUR by adopting open-field, hole cross, swing, and light–dark box tests in Swiss albino mice. We also combined QUR with and/or without a GABA_A_ receptor agonist or antagonist drug to observe possible involvement of GABA_A_ receptors in its anxiolytic effects in experimental animals. Additionally, a computational study was also undertaken to observe the interaction between QUR and GABA receptors for its anxiolytic-like effect in experimental animals.

## 2. Results

### 2.1. Animal Study

#### 2.1.1. Open-Field Test

In the first squad, animals of the negative control (NC) group showed the highest numbers of field cross and rearing. Both DZP (2 mg/kg) and QUR (50 mg/kg) significantly (*p* < 0.05) reduced the test parameters in experimental animals. There is a reduced grooming number in DZP group animals compared to the QUR group. DZP pre-treated with QUR was also found to decrease significantly (*p* < 0.05) in field cross, grooming, and rearing of test animals when compared to the NC and QUR groups. However, the field cross and rearing parameters were higher than in the DZP group.

In the second squad, pre-treatment of FLU (2.5 mg/kg) showed the highest numbers of field cross, grooming, and rearing. FLU combined with DZP resulted in a significant reduction in square crossing and grooming numbers rather than the rearing parameter. On the other hand, FLU, when co-treated with QUR, was found to reduce the number of field cross while reducing other parameters in mice in comparison to the FLU and other treatment groups. The QUR treated with DZP + FLU group resulted in significant alterations in all the test parameters in comparison to the NC, DZP, QUR, and FLU groups (Table 1).

#### 2.1.2. Hole Cross Test

In this study, in the first squad, mice pre-treated with the NC exhibited the highest number of hole cross (25.67 ± 1.37). Both DZP and QUR reduced the hole cross capability in experimental animals as compared to the NC group. QUR combined in the DZP pre-treated group reduced the hole cross significantly (*p* < 0.05) when compared to the NC group; however, it increased the hole cross parameter more than the DZP and QUR groups.

In the second squad, animals pre-treated with FLU (2.5 mg/kg, i.p.) showed maximum hole cross capability. Pre-treatment of DZP or QUR caused a reduction in the hole cross parameter in experimental animals significantly (*p* < 0.05) when compared to the NC and FLU groups. However, QUR in the pre-treatment group of DZP + FLU was found to reduce the test parameter significantly in comparison to the NC, DZP, and QUR groups (Table 2).

#### 2.1.3. Swing Test

In this case, in the first squad, mice pre-treated with the NC produced the highest number of swings (32.07 ± 2.08). Both DZP and QUR reduced the number of swing parameters in experimental animals when compared to the NC group. The QUR plus DZP group also reduced the number of swings significantly (*p* < 0.05) in comparison to the NC group. In the second squad, animals pre-treated with FLU augmented the number of swings in test animals. Both DZP and QUR significantly (*p* < 0.05) reduced the number of swings in experimental animals when compared to the FLU group. QUR combined with DZP + FLU was found to reduce significantly the number of swings in comparison to the NC, DZP, and QUR groups (Table 3).

#### 2.1.4. Light–Dark Test

In this study, DZP significantly (*p* < 0.05) increased the residence time of mice in the lightbox compared to the NC group animals. QUR alone and with DZP also increased the light residence capacity of the animals, where better activity was recorded in the DZP + QUR group (129.00 ± 12.09 s).

FLU also augmented the light residence capacity of the mice. However, FLU co-treated with the DZP or QUR significantly reduced the light residence time in experimental animals. Finally, QUR when combined with the pre-treatment of the DZP + FLU showed a significant (*p* < 0.05) increase in light residence capacity as compared to the NC and DZP groups (Table 4).

### 2.2. In silico Study

#### 2.2.1. GABA Homology Model

Homology modeling has evolved into a valuable structural biology tool, drastically narrowing the gap across empirically observed protein molecules with recognized protein sequences [20]. Employing completely automated platforms and databases, the homology modeling process is streamlined and standardized, permitting especially those without a particular computational foundation to build proper protein mappings and also have rapid and unambiguous referencing to modeling discoveries, visualization, and evaluation [21,22].

**Figure 1:**
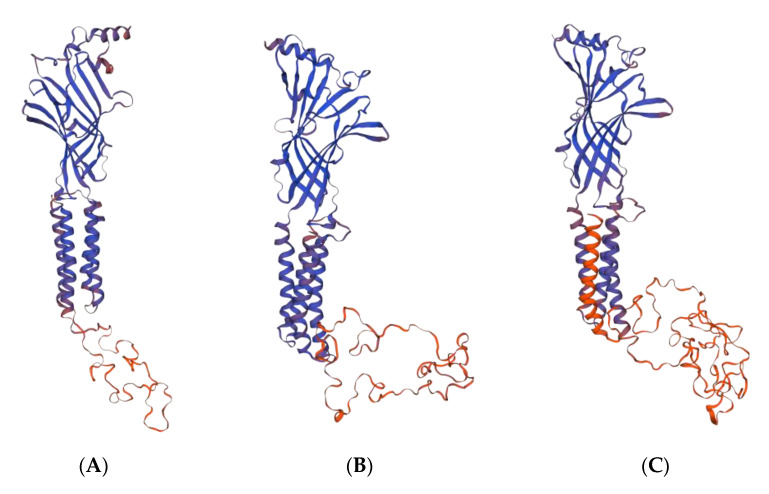
GABA receptor homology model using the Swiss model (**A**) GABA-α5, (**B**) GABA-β1, and (**C**) GABA-β2.

The amino acid sequences of GABA subunits α5, β1, and β2 were obtained from Uniprot (Uniprot accession IDs: P31644, P18505, and P47870, respectively), and NCBI Blast was used to find the most comparable template. The Swiss model was used to create a homology model of GABA (α5, β1, and β2). The 3D homology model of GABA receptors is shown in Figure 1.

**Figure 2:**
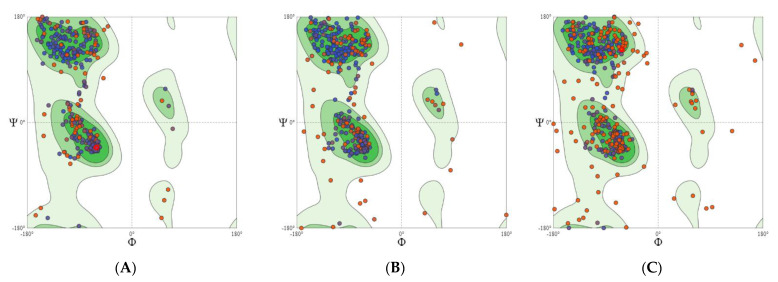
The optimized model of human GABA receptors (**A**) α5, (**B**) β1, and (**C**) β2.

Before docking, the Swiss-PDB Viewer software tool (version 4.1.0) was used to optimize the GABA models. The Ramachandran plot, which was obtained using PROCHECK [3] and depicted in Figure 2, was used to validate these GABA homology models.

The Ramachandran plot is a quick technique to determine how torsion angles are distributed in a protein structure. It also provides the permissible and banned ranges of torsion angle values, which is useful for evaluating the quality of three-dimensional protein structures. The phi-psi (φ-ψ) torsion angles for all residues in the structure are represented by the Ramachandran plot (except those at the chain termini). Because glycine residues are not restricted to plot portions defined for the other side chain types, triangles are utilized to represent them. The coloration and shade of the map reflects the various sections described: the darkest parts (shown in red) correspond to the “core” regions, which represent the most beneficial φ-ψ value combinations. In a perfect world, these “core” sections would contain over 90% of the leftovers. The proportion of residues in the “core” areas is one of the best predictors of stereochemical quality (Figure 2). The residues in the most preferred areas for GABA α5, β1, and β2 are around 92.36%, 92.26%, and 90.8%, respectively, according to Ramachandran plot statistics.

#### 2.2.2. Quercetin (QUR), Diazepam (DZP), and Flumazenil (FLU) with GABA Receptor Interaction

**Figure 3:**
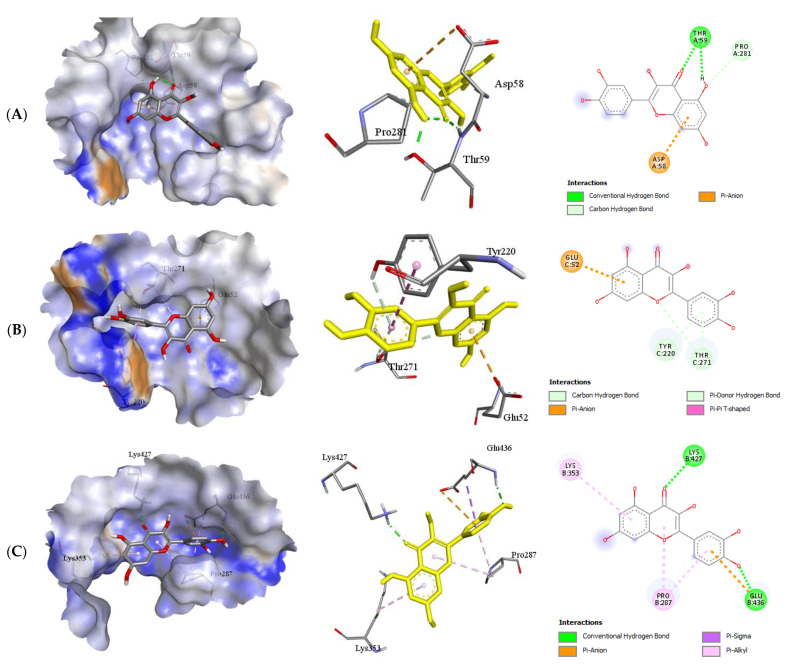
The 2D and 3D structure of molecular docking interactions of (**A**) GABA (α5), (**B**) GABA (β1), and (**C**) GABA (β2) receptor with quercetin (QUR).

QUR exerted high binding affinities for GABA receptor subunits α5, β1, and β2, with −6.8, −8.5, and −8.1 kcal/mol, respectively. Through one H-bond with Thr59 (1.99 Å), one carbon hydrogen, and one pi-anion bond with Pro281 and Asp58, QUR was able to bind to the GABA α (5) subunit. Furthermore, QUR binds to the GABA β1 subunit by two C–H bonds with Thr271 and Tyr220; pi-anion bonds with Glu92. Moreover, QUR bound with GABA β2 subunit through two H-bonds with Glu436 (2.06 Å), Lys427 (2.11 Å), and two pi–alkyl bonds with Lys 353 and Pro287 (Table 5). The 2D and 3D structures of non-bond interactions of QUR with GABA receptor subunits are shown in Figure 3.

**Figure 4:**
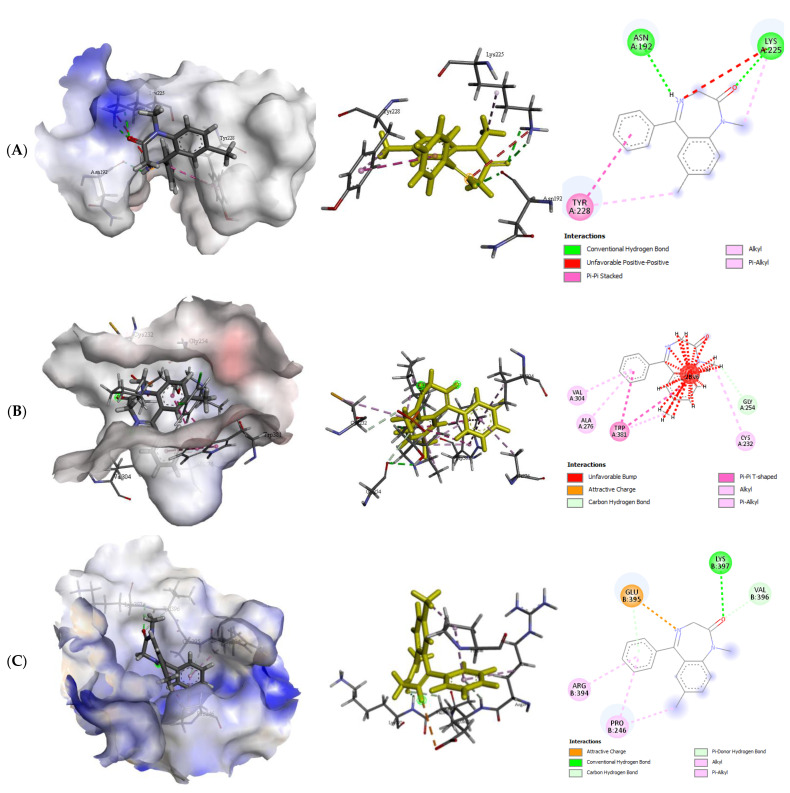
The 2D and 3D structure of molecular docking interactions of (A) GABA (α5), (**B**) *GABA* (β1), and (**C**) GABA (β2) receptor with diazepam (DZP).

DZP, on the other hand, had moderate binding interactions with GABA receptor subunits α5, β1, and β2 at −6.2, −7.0, and −6.4 kcal/mol, respectively. DZP was able to attach to the GABA *α*5 subunit via two H-bonds with Asn192 (2.20) and Lys225 (2.37) and one unfavorable positive–positive and one pi–alkyl bond with Try228. DZP also interacts with the GABA *β*1 subunit via C–H bonds with Gly254 and pi–pi bonds with Trp381, as well as pi–alkyl interactions with Val304, Ala276, and Cys232. DZP also attaches to the GABA *β*2 subunit through one H-bond with Lys397 (2.78), one carbon–hydrogen bond with Val396, and two pi–alkyl bonds with Arg394 and Pro245 (Table 6). Figure 4 depicts the 2D and 3D structures of QUR non-bond interactions with GABA receptor subunits.

**Figure 5:**
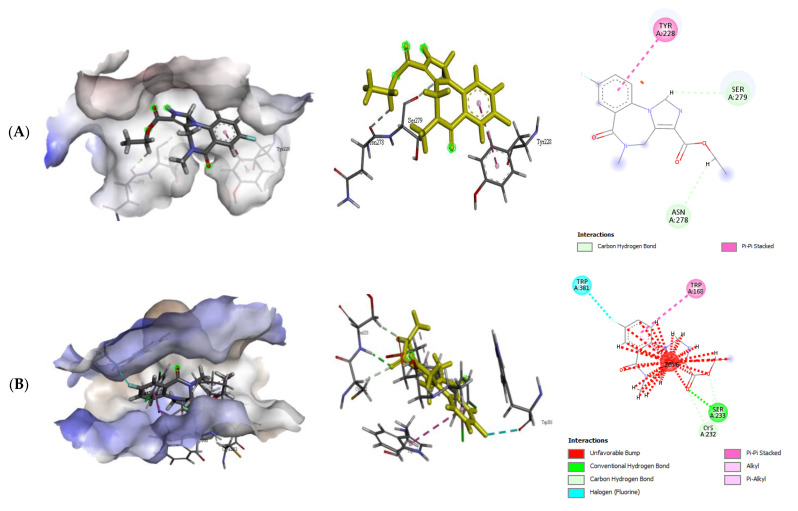

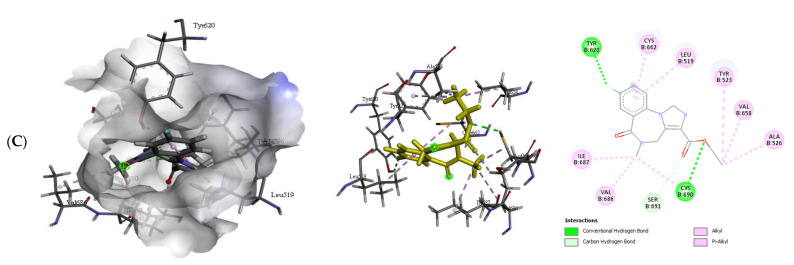
The 2D and 3D structure of molecular docking interactions of (**A**) GABA (α5), (**B**) GABA (β1), and (**C**) GABA (β2) receptor with quercetin (QUR).

Eventually, FLU binds to GABA receptor subunits α5, β1, and β2 with −6.0, −6.4, and −7.9 kcal/mol, respectively, through two carbon–hydrogen bonds and one pi–pi bond, respectively, with Asn278, Tyr228, and Ser 279. FLU may attach to the GABA α5 subunit. Furthermore, FLU interacts with GABA β1 subunit through two H-bonds with Ser233 (2.25 Å) and pi–pi and pi–alkyl bonds with Trp168 and Trp381. Additionally, FLU interacts with GABA β2 subunit through two H-bonds with Cys690 (2.55 Å) and Tyr620 (2.55 Å), one carbon–hydrogen bond with Ser691, and six pi–alkyl bonds with Ala526, Cys662, and ILE68 (Table 7). Figure 5 depicts the 2D and 3D structures of QUR non-bond interactions with GABA receptor subunits.

#### 2.2.3. MD Simulation Study

**Figure 6:**
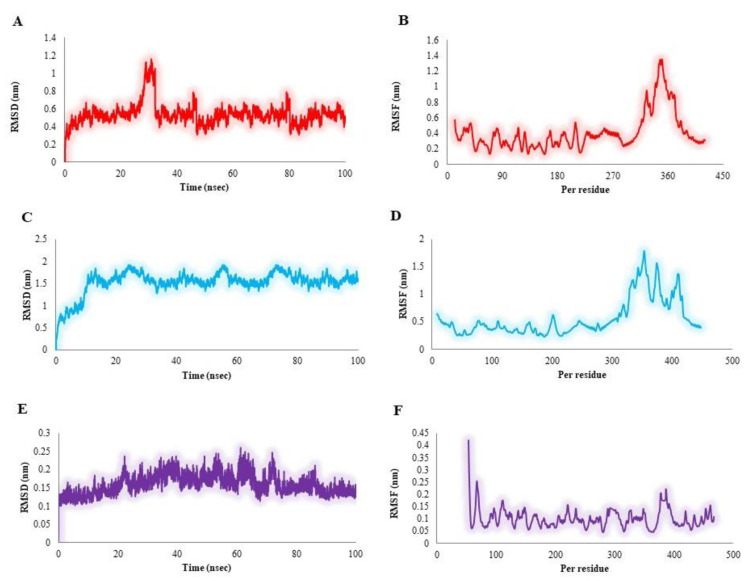
Time evolution of the RMSD values for the complexes of (**A**) backbone atoms of GABA (α5) in QUR-bound GABA (α5) complex, (**B**) residue variation in QUR-bound GABA (α5), (**C**) backbone atoms of GABA (β1) in QUR-bound complex, and (E) backbone atoms of GABA (β2) in QUR-bound complex. (**D**) Residue variation in QUR-bound GABA (β1). RMSF values for the complexes of (**A**) backbone atoms of GABA (α5) in QUR-bound GABA (α5) complex, (**C**) backbone atoms of GABA (β1) in QUR-bound complex, (**E**) backbone atoms of GABA (β2) in QUR-bound complex, and (**F**) residue variation in QUR-bound GABA (β2).

The RMSD values for QUR-bound GABA (α5) GABA (β1) and GABA (β2) proteins were determined and are presented in Figure 6A,C,E), respectively. The RMSD values for protein backbone atoms of MD trajectories for GABA (α5) in QUR-bound form were shown in Figure 6A for the first protein of the selected targets. After 7 ns, the QUR-bound GABA α5 complex achieved equilibrium, and the system displayed steady fluctuation (0.5 nm to 0.9 nm), except for a brief spike in GABA’s RMSD value (α5) and then a return to a stable pattern of fluctuation. The RMSD of backbone atoms of QUR-bound GABA (α5) fluctuated less than the RMSD of backbone atoms of AA-bound GABA (α5) in general. Furthermore, there were three peaks of RMSD variations in the RMSD of backbone atoms of QUR-bound GABA (β1). The RMSD values ranged within 2–1.4 nm during the first 100 ns (Figure 6C). QUR-bound GABA (β2) had a lower RMSD of the backbone than QUR-bound GABA (α5) (Figure 6A) and QUR-bound GABA (β1) (Figure 6C).

The average RMSF of the complete complex backbone is determined during 100 ns and provided in Figure 4 to check the influence of structural flexibility QUR on the rest of the proteins GABA (α5), GABA (β1), and GABA (β2). Figure 6 shows the residue-by-residue variation in QUR-bound GABA (α5), QUR-bound GABA (β1), and QUR-bound GABA (β2) (Figure 6B,D,F, respectively).

#### 2.2.4. Binding Free Energy (MM-PBSA) Analysis

The MM-PBSA program provides several individual components, such as G_vdW_, ∆G_elec_, ∆G_pol_, and ∆G_nonpol_, to compute the total binding free energy (∆E MMPBSA), which is utilized to understand the biophysical foundation for molecular recognition of AA and QUR with targets GABA(α5), GABA(β1), and GABA(β2). The intermolecular van der Waals interaction (∆G_vdW_), electrostatic interaction (∆G_elec_), and solvation energy are all non-polar (∆G_nonpol_), and the promotion of free polar solvation energy (∆G_pol_) is unfavorable, as shown in Table 8. Table 8 shows that GABA α5–QUR has the greatest estimated interaction free energy (∆E MM-PBSA) (−27.071 kJ/mol) despite unfavorable contributions from ∆G_pol_ (83.475 kJ/mol). The polar contribution to the GABA β1–QUR complex has the largest positive value (∆G_pol_ = 145.703 kJ/mol). GABA α5 had a stronger interaction with QUR than GABA β1 and GABA β2. However, QUR’s binding affinities with specific proteins are often high.

## 3. Discussion

Flavonoids are plant secondary metabolites that can have a multitude of different bio-pharmacological effects, notably anti-oxidative and anti-inflammatory properties [23]. According to preliminary studies, QUR is moderately poisonous to rats, highlighting the importance of this research because this chemical is found in a significant number of medicinal plants now used by people all over the world [24]. In a recent study, QUR was found to produce anti-anxiety and anti-depressant effects, along with enhancement in memory capacity in mice, possibly through preventing impairment in antioxidant enzymes and regulation of the serotonergic and cholinergic neurotransmission pathways [17]. QUR (50 mg/kg) was observed to suppress adrenocorticotropic hormone (ACTH) and corticosterone levels in another study on mice [15].

QUR had first been assessed using an open-field test, which also provides a good indication of experimental animals’ exploratory activity [25]. DZP has long been used as a conventional anxiolytic, as well as a reference compound for a significant anxiolytic-acting compound in behavioral pharmacology [26,27].

In this study, QUR was found to augment the calming effects of DZP while reducing the effects of FLU in experimental animals. However, in comparison to the DZP group, QUR moderately increases the number of crossings and rearing parameters in the test animals. Probably, QUR has an anxiolytic effect on the test animals unrelated to motor coordination or neuromuscular blockade; instead, it may be caused by central depressant activity.

Animals with normal movements frequently pass through the hole inside the hole board box. Similarly, the movement of the test animal inside the swing box causes the swing of the box [28]. Any substance that can affect normal neurological activity can alter the movement inside these boxes.

GABA is the chief inhibitory neurotransmitter in the mature mammalian central nervous system (CNS). It reduces neuronal excitability throughout the nervous system [29]. DZP augments the levels of GABA in CNS [30]. It is a medicine of the benzodiazepine class that typically produces a calming effect in animals [31].

In our study, DZP resulted in a reduction in the number of hole crosses and swings, suggesting a calming effect in experimental animals. QUR alone or with the DZP group was observed to reduce the number of the same parameters in the hole-board and swing tests when compared to the QUR group. FLU antagonizes the action of DZP by exerting its effects through the same receptor [32].

Our findings also suggest an opposite effect of DZP for the FLU-treated animals, which was confirmed by the increased number of hole crosses and swings in mice. Further, the co-treatment of DZP and/or QUR with FLU was observed to decrease the test parameters in comparison to the FLU group in these test models: QUR produced a moderate flumazenil (FLU)-like effect in the test animals, and a number of studies have demonstrated that QUR binds with GABAA and B receptors but exerts an antagonistic effect similar to FLU [33,34]. Therefore, when DZP was administered with QUR, QUR exposed a calming effect.

The conflict between the desire to explore and the desire to retreat from an unfamiliar and well-lit space generates anxiety in the light–dark box test [35]. In this study, both DZP and QUR were found to increase the light residence time in the study, confirming the anxiolytic-like effect of these compounds. QUR alone or its combination with DZP significantly increased the light residence time of the test animals.

Interestingly, QUR augmented light residence time more than the DZP group, suggesting its potential calming effects in mice. Moreover, in combination with the DZP + FLU group, QUR showed the highest increase in light residence time. Since FLU is a GABA-A-receptor-selective inhibitor at the BDZ active site, which has the potential to suppress anxiety, it is possible that QUR can engage with the GABA A receptor subunits essential for anxiolytic activity [36]. Moghbelinejad et al. also suggested that QUR mediated anti-seizure effects in experimental animals, possibly through an interaction with GABAA α5 receptor [18].

Thus, our study agrees with the QUR-mediated calming effects in Swiss mice, possibly through the GABAA receptor interaction pathway. Anxiolytic effects are mediated by GABAα2 receptors, while α3 and α5 contribute to the myorelaxant actions of DZP [37]. FLU also binds to GABAA to induce its anxiolytic effects [20].

**Figure 7:**
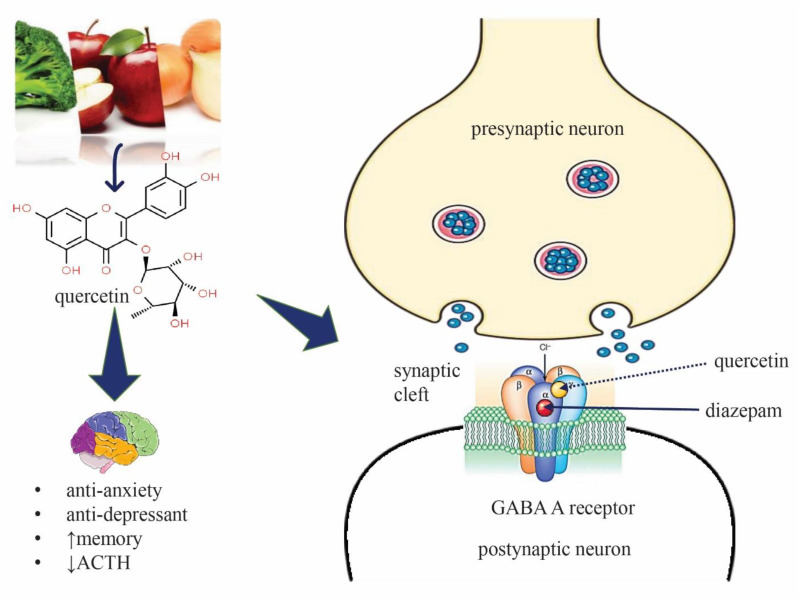
Anxiolytic-like effect and potential molecular mechanism of action of quercetin with GABA A receptor. Legend: ↑ increase; ↓ decrease; the arrow with a solid line shows that diazepam binds GABAA α2, whereas the arrow with a dotted line suggests that QUR’s anti-anxiety action might be linked to potential regulation of GABA A α2 subunit; ACTH, adrenocorticotropic hormone.

QUR may exert its anxiolytic-like effects through GABAA receptors containing α2 interaction pathways. The findings of our in silico study also suggest that it has good interaction capability with the GABA receptor, especially with its α2, β1, and β2 subunits. However, a potential interaction was observed with the GABAα2 subunits. Considering the in vivo and in silico studies, it is possible to draw a possible anxiolytic effect pathway of QUR in Figure 7.

## 4. Materials and Methods

### 4.1. In Vivo (Animal) Study

#### 4.1.1. Chemicals and Reagents

Quercetin (QUR) was purchased from Merck (India). The sources of diazepam (DZP) (Sedil) and flumazenil (FLU) (Anexate) were Square Pharmaceutical Ltd., Dhaka, Bangladesh, and Roache Pharmaceuticals Ltd., Switzerland, respectively.

#### 4.1.2. Animal Model

This study utilized male adult Swiss albino mice (22–30 gm) bought from the veterinary supply section of Jahangir Nagar University (JU), Dhaka. The mice were allowed in sterilized polypropylene cages with husk covering under normal weather conditions (temperature: 25 ± 2 °C, humidity: 50 5%, and 12 h light/dark cycles). The mice had unlimited access to conventional granules as a base diet and ad libitum water. Before beginning the research, all the mice were given a two-week acclimatization period. The Institutional Animal Care and Use Committee of the Life Science Faculty, Bangabandhu Sheikh Mujibur Rahman Science and Technology University (Approval No. 20151109011), Gopalganj, Bangladesh authorized all procedures in line with the Guide for the Care and Use of Laboratory Animals.

#### 4.1.3. Study Design

Experimental animals were deprived of food 6 h before the test commenced. Then, the animals were randomized into experimental and control groups, each containing 5 animals. Briefly, the animals were divided into eight groups denoted as Gr.-I to Gr.-VIII.

The groups receiving treatment(s) are shown in Table 9. Each group received negative control, positive control, and the different drugs and drug combinations. The dosages of the sample material and control drugs were modified based on the weight of each mouse.

All the treatments (QUR, DZP, and FLU) were reconstituted in the vehicle. The first three groups (i.e., Gr.-I, Gr.-II, Gr.-III) were treated with the vehicle (10 mL/kg), diazepam (DZP, 2 mg/kg), and quercetin (QUR, 50 mg/kg), respectively. The animals were given medication 45 min after they were subjected to the open board, hole board, swing apparatus, and light–dark box tests. To evaluate the combined effects of QUR with DZP (Gr.-IV), 5 animals were pre-treated with QUR (5 mg/kg) 15 min before DZP (2 mg/kg) administration.

To evaluate if the QUR effect was mediated by GABA/benzodiazepine receptor, four groups (Gr.-V to Gr.-VIII) of mice were treated with flumazenil (FLU, 2.5 mg/kg) with or without DZP and/or QUR and the test parameters of each animal model were verified similar to protocol performed previously. All the treatments were administered via the intraperitoneal route.

#### 4.1.4. Experimental Protocol

The animals were examined in a confined chamber with a comfortable temperature of 26 2 ^◦^C during light time (09.00 am to 12.00 pm). All the experiments were carried out on separate days with various animal groups. 

#### 4.1.5. Open-Field Test

We utilized a wood open-field area with a drawn square (split into nine squares of equal area) floor (30 × 30 × 30 cm^3^) in this test. This device was utilized to assess the animals’ inquisitive activities throughout 5 min using the methodology provided [38,39]. For each mouse, the number of squares traversed with the four paws (spontaneous locomotor activity), grooming behavior (grooming), and surveys (rearing) were documented. The ground was wiped using 70% ethyl alcohol after each experiment.

#### 4.1.6. Hole Cross Test

The procedure was followed exactly as instructed in Ref. [40]. We utilized a wooden barrier installed in the middle of a cage with a size of 30 × 20 × 14 cm^3^ in this experiment. In the lowest portion of the cage’s dividing board, a 3-cm-diameter hole was drilled. The experiment was conducted according to instructions of Subhan et al. [41]. Each mouse was instantly put on one end of the hole-board device after a 3-min open-field test. For 5 min, the mice were seen moving freely from one room to the next via the hole. Each test was followed by thorough cleaning of the equipment’s floor.

#### 4.1.7. Swing Test

The apparatus consisted of a 120 gm PP (polypropylene)-made swing box (21.5 × 12.5 × 11.5 cm) mounted on a swing rod (42.5 × 1.5 cm). The whole setup was infrastructure on a stage (36.5 × 29 × 2 cm). A supporting stand was connected towards the holding stands. The swing rod, stage, supporting, and holding stands were made of wood. The lower portion of the swing box bisectionally (equally) was fitted with a swing rod with the help of a stainless-steel rod. The experimental animals after administration of the treatments under investigation were placed inside the swing box and the number of swings due to frequent movements of each animal inside the swing box was recorded. After each test, the floor of the swing box was cleaned with 70% ethanol [28].

#### 4.1.8. Light–Dark Test

The research equipment is composed of wood and is separated into two chambers (5 light box and dark box) that are connected by a tiny door [42,43]. The dark box is dimly lighted (black portion: 271,829 cm^3^), whereas the light box (27 × 18 × 29 cm^3^) is lit by ambient light. Each mouse was instantly put on one side of the light–dark apparatus after a 3-min swing test. Using a stopwatch, the time spent (sec) in the dark and light portions for each animal was recorded over 5 min. Each test was followed by a thorough cleaning of the equipment’s floor.

**Scheme 1:**
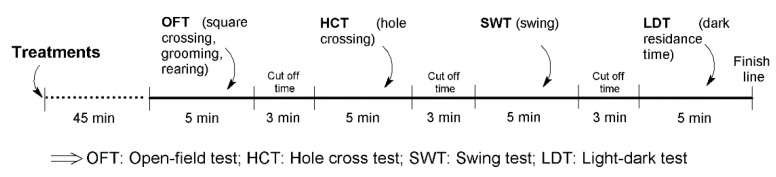
Study design for the anxiolytic test in Swiss albino mice.

Scheme 1 presents the protocol for this study.

#### 4.1.9. Statistical Analysis

The mean and standard error of the mean are used to express all values (SEM). GraphPad Prism software (version: 6.0, San Diego, CA, USA. Copyright 1994–1999) was used to analyze the data using analysis of variance (ANOVA), followed by *t*-Student–Neuman–Keuls post hoc test, with *p* < 0.05 at a 95 percent confidence interval.

### 4.2. Molecular Docking (In Silico) Study

#### 4.2.1. Protein and Ligand Preparation

**Figure 8:**
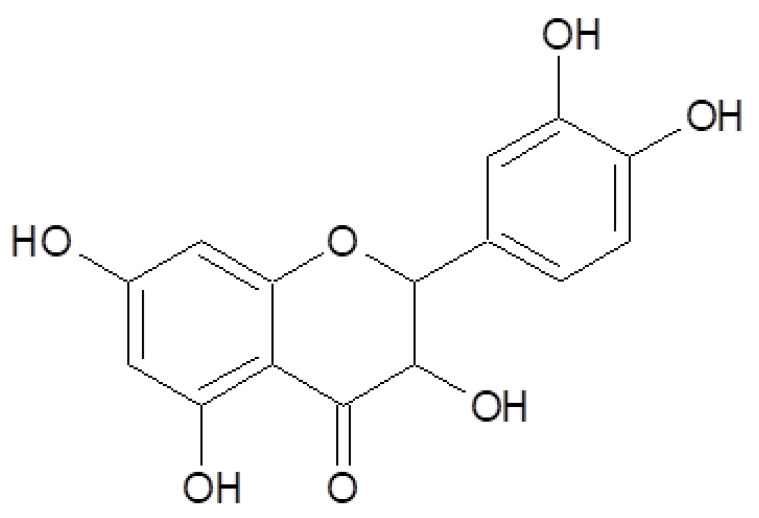
Chemical structure of quercetin.

The Swiss model [22] was used for homology model of human gamma-aminobutyric acid (GABA). The sequence was obtained from UniProt [4], then BLAST assessment was conducted using the NCBI BLAST [27] tool to choose the template. Evaluation of the homology model was conducted with Ramachandran plot performed by Procheck [3]. The interaction mechanism of GABA was investigated using molecular docking of QUR compounds. In addition, the ‘sdf’ file format was used to acquire the chemical structure of quercetin (QUR) (PubChem ID: 5280343) (Figure 8). Chem3D Pro12.0 program packages [17] were used to optimize all internal energies of the ligands.

#### 4.2.2. Docking Protocol

In medicinal chemistry, molecular docking is a computer tool for drug design. Auto Dock Vina technology was used to evaluate and position medicinal agents to receptor binding sites [23] in order to determine their pharmacodynamic properties. Docked results indicate the level of ligand interaction with the designated protein’s binding site. PyMol and Drug Discovery Studio version 4.5 were utilized to scrutinize these active binding sites of target protein [5]. The active sites correlate to the ligand’s placements in the original target protein grids [9].

#### 4.2.3. Molecular Dynamic (MD) Simulation and MM-PBSA Study

Molecular dynamic simulation is an important tool for assessing the binding affinity of small molecules at the active site and analyzing the stabilization of the complex [41]. A time axis shows conformational changes or behaviors that can be used to determine if the target ligand molecule is stable.

The selected protein–QUR complexes were subjected to MD simulations in the current study. To execute MD simulations, we used the GROMACS 2020.1 software package [40]. The Swiss Param web-server [1] was used to produce the parameters and topology of QUR, whereas Charm 27 forcefield [37] was employed to parameterize the 3D structure of proteins. To dissolve the protein–QUR complexes, use a solvent with three-point transfer of intermolecular potential (TIP3P) [6]. The protein–QUR system can be neutralized with salt and chloride ions if necessary. The steepest descent approach is then used to reduce the energy of protein–drug systems to a tolerance threshold of 1000 kj/mol.nm. Then, using NVT and NPT ensembles, use equilibration with the role of position restraint at the protein molecules for 0.1 ns. To examine all the electrostatic interactions of biological systems, Particle Mesh Ewald (PME) was used. The next step is to conduct MD simulations without any protein or amygdalin molecules in the way. Finally, MD simulations were run on a 100 ns time scale (3 fs time step).

We estimated several features using a special technique based on the MD simulation results, including the root-mean-square deviation (RMSD) using gmx RMS and the root-mean-square fluctuation of residues (RMSD) using gmx RMSF.

Molecular mechanics/Poisson–Boltzmann surface area was employed in addition to molecular dynamics to estimate the thermodynamic stability of QUR at the target binding site and to test the overall binding affinities of QUR with selected targets. The g_mmpbsa [29] script tool was used to perform the calculations. This approach is based on the average of two energy values: the solvation energy and the potential energy in vacuum.

∆E (MM − PBSA) = ∆EMM + ∆G_solvation._ (1)

In Equation (1), EMM and G_solvation_ are the vacuum potential energy and free solvation energy, respectively. The electrostatic component (E_ele_) and the van der Waals interaction (EvdW) are used to calculate the molecular mechanical energy (EMM). The polar solvation energy G_pol_ and the non-polar solvation energy G_nonpol_ are used to compute the solvation energy. The Poisson–Boltzmann equation (PB) is used to determine G_pol_, and the solvent-accessible surface is used to calculate G_nonpol_ (SASA). For the last 20 ns of MD trajectories, MM-PBSA computations were performed.

## 5. Overall Conclusions and Future Perspectives

Our findings show that acute QUR treatment has an anxiolytic effect on Swiss mice. Additionally, the findings support the hypothesis that QUR interactions with both the GABAA and GABAB receptors provide an anxiolytic (calming or tranquilizing) effect on this system model, most likely at the receptor subtypes that generate benzodiazepine actions. QUR may cause anxiolytic-like effects in Swiss mice, potentially via regulation of GABA receptors, particularly activation with GABA α5 and GABA β1 receptors, based on our preclinical experimental and molecular docking studies.

## Figures and Tables

**Table 1:** Effects of test sample and controls in Swiss mice (open-field test).

Treatment Groups	Number of Squares Crossed	Number of Grooming	Number of Rearing
Vehicle	49.13 ± 2.56	2.67 ± 0.44	35.17 ± 1.85
DZP	23.60 ± 2.03 ^a^	2.00 ± 0.36 ^a^	19.20 ± 7.92 ^a^
QUR	42.80 ± 3.80 ^a^	2.60 ± 0.76	27.80 ± 6.83 ^a^
DZP + QUR	30.40 ± 1.30 ^ac^	2.00 ± 0.80 ^ac^	22.00 ± 10.30 ^ac^
FLU	50.75 ± 0.71	3.27 ± 0.52	39.78 ± 1.09
DZP + FLU	49.97 ± 1.20 ^bc^	1.57 ± 0.88 ^abc^	40.09 ± 1.41 ^abc^
QUR + FLU	47.00 ± 3.30 ^bc^	3.43 ± 0.35 ^abc^	43.00 ± 2.98 ^abc^
DZP + FLU + QUR	28.8 ± 2.87 ^abc^	2.40 ± 0.57 ^abc^	15.60 ± 2.14 ^abc^

Values are mean ± SEM (*n* = 5) (ANOVA followed by *t*-Student–Neuman–Keuls post hoc test); ^a^ *p* < 0.05, significantly different from negative control (NC) group (vehicle); ^b^ *p* < 0.05, significantly different from diazepam (DZP) group; ^c^ *p* <0.05, significantly different from quercetin (QUR) group; FLU: flumazenil.

**Table 2:** Effects of test sample and controls in Swiss mice (hole cross test).

Treatment Groups	Number of Hole Cross
Vehicle	25.67 ± 1.37
DZP	15.80 ± 3.86 ^a^
QUR	17.03 ± 1.27 ^a^
DZP + QUR	18.01 ± 2.15 ^ab^
FLU	26.07 ± 1.08
DZP + FLU	20.67 ± 2.61 ^abc^
QUR + FLU	22.00 ± 1.37 ^abc^
DZP + FLU + QUR	12.60 ± 2.41 ^abc^

Values are mean ± SEM (*n* = 5) (ANOVA followed by *t*-Student–Neuman–Keuls post hoc test); ^a^ *p* < 0.05, significantly different from negative control (NC) group (vehicle); ^b^ *p* < 0.05, significantly different from diazepam (DZP) group; ^c^ *p* < 0.05, significantly different from quercetin (QUR) group; FLU: flumazenil.

**Table 3:** Effects of test sample and controls in Swiss mice (swing test).

Treatment Groups	Number of Swings
Vehicle	32.07 ± 2.08
DZP	19.60 ± 3.73 ^a^
QUR	22.40 ± 2.56 ^a^
DZP + QUR	24.00 ± 2.45 ^a^
FLU	33.08 ± 2.78
DZP + FLU	30.21 ± 2.54 ^abc^
QUR + FLU	29.60 ± 1.21 ^bc^
DZP + FLU + QUR	16.60 ± 2.80 ^abc^

Values are mean ± SEM (*n* = 5) (ANOVA followed by *t*-Student–Neuman–Keuls post hoc test); ^a^ *p* < 0.05, significantly different from negative control (NC) group (vehicle); ^b^ *p* < 0.05, significantly different from diazepam (DZP) group; ^c^ *p* < 0.05, significantly different from quercetin (QUR) group; FLU: flumazenil.

**Table 4:** The light residence time of the test animals pre-treated with the sample and controls (light–dark test).

Treatment Groups	Time Spent in Light Box (s)
Vehicle	104.20 ± 1.48
DZP	118.60 ± 2.92 ^a^
QUR	126.20 ± 4.27 ^ab^
DZP + QUR	129.00 ± 2.09 ^abc^
FLU	113.30 ± 1.01 ^a^
DZP + FLU	106.50 ± 0.65 ^a^
QUR + FLU	104.60 ± 1.08
DZP + FLU + QUR	122.00 ± 4.67 ^ab^

Values are mean ± SEM (*n* = 5) (ANOVA followed by *t*-Student–Neuman–Keuls post hoc test); ^a^ *p* < 0.05, significantly different from negative control (NC) group (vehicle); ^b^ *p* < 0.05, significantly different from diazepam (DZP) group; ^c^ *p* < 0.05, significantly different from quercetin (QUR) group; FLU: flumazenil.

**Table 5:** The best three results of molecular docking study of quercetin (QUR) with GABA receptor subunits.

Protein (Receptor)	Binding Affinity (Kcal/mol)	No. of H-Bond	H-Bond Residues	H-Bond Length (Å)	Other Bond Residues
GABA α5–QUR	−6.8	1	Thr59	1.99	Asp58 Pro281
GABA β1–QUR	−8.5	0	-	-	Glu92 Thr271 Tyr220
GABA β2–QUR	−8.1	2	Glu436 Lys427	2.06 2.11	Lys 353 Pro287

**Table 6:** The best three results of molecular docking study of diazepam (DZP) with GABA receptor subunits.

Protein (Receptor)	Binding Affinity (Kcal/mol)	No. of H-Bond	H-Bond Residues	Bond Length (Å)	Other Bond Residues
GABA α5-DZP	−6.2	2	Asn192 Lys225	2.20 2.37	Try228
GABA β1-DZP	−7.0	0	-	-	Ala276, Cys232, Gly254, Trp381, val304
GABA β2-DZP	−6.4	1	Lys397	2.78	Arg394, Glu395, Pro245, Val396

**Table 7:** The best three results of molecular docking study of flumazenil (FLU) with GABA receptor subunits.

Protein (Receptor)	Binding Affinity (Kcal/mol)	No. of H-Bond	H-Bond Residues	Bond Length (Å)	Other Bond Residues
GABA α5-FLU	−6.0	0	-	-	Asn278, Tyr228, Ser 279
GABA β1-FLU	−6.4	1	Ser233	2.25	Cys232, Trp168, Trp381
GABA β2-FLU	−7.9	2	Cys690 Tyr620	2.55 2.55	Ala526, Cys662, ILE687, Leu519, Ser691, Val 658, Val686

**Table 8:** The calculated binding energies of AA and QUR/targets (GABA(α5), GABA(β1), and GABA(β2)) complexes.

Complex Name	∆G_vdW_ (kJ/mol)	∆G_elec_ (kJ/mol)	∆G_pol_ (kJ/mol)	∆G_nonpol_ (kJ/mol)	∆E (MM-PBSA) (kJ/mol)
GABA α5–QUR	−59.798	−39.976	83.475	−8.699	−27.071
GABA β1–QUR	−75.562	−80.536	145.703	−13.908	−25.083
GABA β2–QUR	−83.053	−54.634	127.317	−11.403	−16.850

**Table 9:** Groups treated intraperitoneally in Swiss mice (n = 5).

Treatment Groups	Description	Dose
Gr.-I: Negative control	Vehicle 0.5% tween 80 + 0.9% NaCl solution	10 mL/kg
Gr.-II: DZP	Diazepam Standard 1: Benzodiazepine receptor agonist	2 mg/kg
Gr.-III: QUR	Quercetin Test sample	50 mg/kg
Gr.-IV: DZP + QUR	Diazepam + Quercetin	2 mg/kg + 50 mg/kg
Gr.-V: FLU	Flumazenil Standard 2: Benzodiazepine receptor antagonist	2.5 mg/kg
Gr.-V-I: DZP + FLU	Diazepam + Flumazenil	2 mg/kg + 2.5 mg/kg
Gr.-VII: QUR + FLU	Quercetin + Flumazenil	50 mg/kg + 2.5 mg/kg
Gr.-VIII: DZP + FLU + QUR	Diazepam + Flumazenil + Quercetin	2 mg/kg + 2.5 mg/kg + 50 mg/kg

## Data Availability

The processed data are available from the corresponding author upon request.
